# Exploring the causal factor effects of hypothyroidism on ischemic stroke: a two-sample Mendelian randomization study

**DOI:** 10.3389/fneur.2024.1322472

**Published:** 2024-01-31

**Authors:** Yi Tian, Xiao Qin Shi, Jing Wen Shui, Xiao Yu Liu, Ya Bu, Yi Liu, Li Ping Yin

**Affiliations:** ^1^School of Clinical Medicine, Chengdu University of Traditional Chinese Medicine, Chengdu, China; ^2^Department of Communication Sciences and Disorders, MGH Institute of Health Professions, Boston, MA, United States

**Keywords:** hypothyroidism, stroke, ischemic stroke, cerebral infarction, Mendelian randomization analysis

## Abstract

**Background:**

Observational studies have suggested a possible association between hypothyroidism and increased risk of ischemic stroke. However, a causal relationship remains unclear.

**Methods:**

Data on single nucleotide polymorphisms (SNPs) associated with hypothyroidism and ischemic stroke were sourced from the FinnGens database and the UK Biobank of European descent. Both databases underwent separate two-sample Mendelian randomization (MR) analyses. A subsequent meta-analysis of MR results using a random-effects model was conducted to determine the causal relationship between hypothyroidism and ischemic stroke.

**Results:**

All five analyses indicated a positive causal relationship between hypothyroidism and ischemic stroke. MR analysis of the association between hypothyroidism and ischemic stroke yielded a result of the inverse variance weighted (IVW) method at 4.7411 (1.3598–16.5308), *p* = 0.0146. The analysis of ischemic stroke (without excluding controls) yielded a result of the IVW method of 4.5713 (1.3570–15.3986), *p* = 0.0142. MR analysis with cerebral infarction yielded a result of the IVW method at 1.0110 (1.0006–1.0215), *p* = 0.0373. The MR analysis with cerebrovascular disease sequelae yielded an IVW method result of 2.4556 (1.0291–5.8595), *p* = 0.0429. Analysis for the sequelae of cerebrovascular disease (without excluding controls) yielded an IVW method result of 2.4217 (1.0217–5.7402), *p* = 0.0446. No evidence of heterogeneity or horizontal pleiotropy was found. The meta-analysis of the five MR results was 2.24 (1.18–4.26), *p* = 0.025.

**Conclusion:**

Our two-sample Mendelian randomization study suggested a causal relationship between hypothyroidism and ischemic stroke, indicating that hypothyroidism could be a risk factor for ischemic stroke. However, further studies are required to elucidate the underlying biological mechanisms.

## Introduction

Stroke is the second leading cause of global mortality and the third leading cause of severe disability (1). In 2019, ischemic strokes accounted for a significant proportion of documented stroke incidents, resulting in an estimated 50,000 mortalities and a decline of approximately 910.2 million disability-adjusted life years (DALYs). Low-income countries bear a disproportionately larger burden of this disease compared to high-income countries (1–4). IS caused by arterial occlusion is the cause of most strokes. Given that rapid reperfusion such as, endovascular therapy (EVT) and intravenous thrombolysis, is an effective treatment for neurological injuries induced by acute ischemic stroke (AIS), timely and accurate identification of ischemic injuries is paramount for implementing acute interventions and devising strategies to prevent recurrence. Research indicates that both absolute and relative deficiencies in thyroid hormones can intensify cerebrovascular atherosclerosis, thereby influencing the prevalence and outcomes of cerebrovascular diseases (5–7). Some cross-sectional studies have identified a correlation between the concentration of thyroid-stimulating hormone (TSH) and the intensification of symptoms associated with AIS. Compared with euthyroidism, higher incidence of stroke was associated with high TSH or low free T4 levels (TSH >5.5 mIU/L, free T4 < 0.7 ng/dL) (8). Interestingly, this correlation was not observed in the patients with normal thyroid function. The differential effects of thyroid hormone levels across diverse patient groups suggest a multifaceted interaction between hypothyroidism and ischemic stroke. However, such associations observed in observational studies could be influenced by confounding factors and potential biases in the data collection. Thus, a clearer understanding of this potential causal relationship is pivotal for advancing the prevention and management strategies for ischemic stroke.

Using aggregated data from genome-wide association studies (GWAS), we performed two-sample mendelian randomization (MR) analysis to investigate the association between hypothyroidism and ischemic stroke. Rooted in human genetics, this approach utilizes single nucleotide polymorphisms (SNPs) as instrumental variables (IVs) (9). This method employs SNPs as IVs (9), which, in the context of GWAS, are associated with exposure phenomena (9). The MR analysis approach aims to clarify the causal relationship between exposure and outcomes, minimizing the biases present in traditional randomized controlled trials (10).

## Materials and methods

### Study design

**Figure 1:**
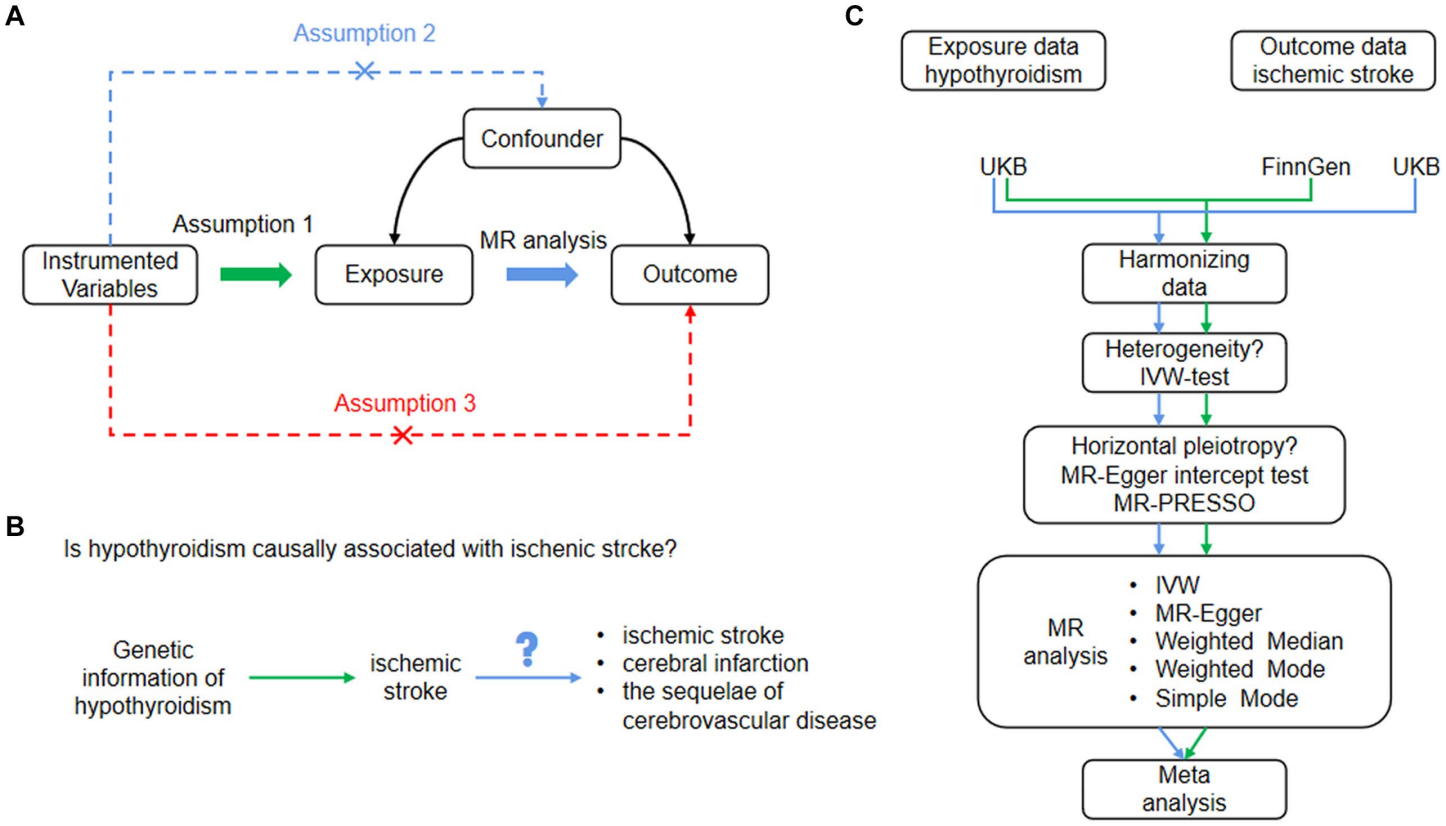
**(A)** Basic assumptions of Mendelian randomization. Assumption 1: SNPs were closely associated with exposure. Assumption 2: SNPs were not associated with any potential confounders. Assumption 3: SNPs are only linked to the outcome through exposure. **(B)** The study design of our MR analysis. **(C)** Flowchart of the study process. IVW, inverse variance weighted; MR, Mendelian randomization; MR-Egger, MR-Egger regression, SNPs, single nucleotide polymorphisms.

In this study, we utilized two-sample MR analysis to investigate the relationship between hypothyroidism (exposure) and ischemic stroke (outcome) using SNPs as IVs. To ensure the reliability of MR analysis, three critical assumptions must be met: (1) SNPs are significantly associated with exposure; that is, hypothyroidism. (2) The SNPs were not associated with any potential confounders. (3) SNPs influenced ischemic stroke risk solely via their association with hypothyroidism. We determined the causal link between hypothyroidism and ischemic stroke using distinct outcome databases in the MR analysis. A subsequent meta-analysis provided a consolidated estimate, as illustrated in Figure 1.

### Data sources

We sourced genetic summary data from two prominent GWAS databases, the FinnGens database and UK Biobank (UKB), as detailed in Table 1. From the UKB,^1^ we procured hypothyroidism-related GWAS data (inquiry code: ukb-b-19732), which comprised of 462,933 participants of European descent, covering a total of 9,851,867 SNPs. Additionally, the cerebral infarction GWAS summary statistics (inquiry code: ukb-d-I63) from the UKB included 361,194 participants of European origin and 10,889,323 SNPs. The FinnGens database,^2^ we accessed two GWAS datasets on ischemic stroke. The first (inquiry code: finn-b-I9_STR_EXH) involved 212,774 individuals and contained 16,380,445 SNPs, whereas the second (inquiry code: finn-b-I9_STR_EXH_EXNONE) consisted of 218,792 individuals and covered 16,380,466 SNPs. Two additional datasets related to cerebrovascular disease sequelae from FinnGens include the first (inquiry code: finn-b-I9_SEQULAE) with 207,706 individuals and 16,380,409 SNPs, and the second (inquiry code: finn-b-I9_SEQULAE_EXNONE) with 218,792 individuals and 16,380,466 SNPs. For all datasets, we gathered details on several SNPs, including effect frequency (EAF), effect size (β), effect allele (EA), and *p*-value. As all the data were publicly accessible, no additional ethical clearance was required. The genetic background of all study participants was of European origin, minimizing the potential bias from ethnic confounding factors.

**Table 1:** Characteristics of data sources used in the Mendelian randomization study.

Exposures/outcomes	Consortium	Ethnicity	Sample sizes	Experimental group	Control group	NSNP
Hypothyroidism	UK Biobank	European	462,933	22,687	440,246	9,851,867
Ischemic stroke	FinnGen Biobank	European	212,774	10,551	202,223	16,380,445
Ischemic stroke (no controls excluded)	FinnGen Biobank	European	218,792	10,551	208,241	16,380,466
Cerebral infarction	UK Biobank	European	361,194	2,353	358,841	10,889,323
Cerebrovascular disease sequelae	FinnGen Biobank	European	207,706	4,638	203,068	16,380,409
Cerebrovascular disease sequelae (without excluding controls)	FinnGen Biobank	European	218,792	4,638	214,154	16,380,466

NSNP, the number of SNPs included in the MR analysis. MR, Mendelian randomization; SNPs, single nucleotide polymorphisms.

### Selection of instrument variables

First, genetic polymorphisms that were substantially correlated with hypothyroidism on a genome-wide scale (*p* < 5 × 10^−10^) were employed as IVs. To ensure the independence of these IVs and eliminate potential bias from linkage disequilibrium (LD), we set an r-squared threshold of less than 0.0001. This meticulous approach effectively restricted the genetic distance to a maximum of 10,000 kilobases (kb), thereby enhancing the robustness and precision of our methodology. Subsequently, SNPs that exhibited a significant correlation with exposure elements were screened using the *F*-statistic of SNPs, and strong IVs were recognized by satisfying *F* > 10. Information on hypothyroidism and ischemic stroke outcomes was extracted using GWAS to determine the relationship between SNPs satisfying Hypotheses 1, 2, and 3 and outcomes. Next, the data extracted from the two databases of exposure and outcome variables were combined and collated, and palindromic sequences were removed to ensure that all SNPs were the same effector allele.

### Two-sample Mendelian randomization analysis

To explore the potential relationships between the specified variables, we conducted a two-sample MR analysis using five analytical techniques: inverse variance weighted (IVW), MR-Egger regression, weighted median (WME) (11), simple mode, and the weighted mode (11). Our primary approach, the IVW method, assessed the direct association between hypothyroidism and ischemic stroke. Cochran’s *Q* test was used to evaluate the heterogeneity among singular genetic variance estimations. When the *p*-value from Cochran’s *Q* test was below 0.05, we adopted the random effects model within IVW for the final MR analysis; otherwise, the fixed-effects model was selected (12). Outcomes in the study were presented using odds ratios (OR) and 95% confidence intervals (CI), constituting the statistical framework for interpretation. The establishment of a *p*-value threshold of less than 0.05 served as the demarcation line, delineating statistically significant differences from those that fall within the realm of statistical insignificance. We visualized our results using scatter and forest plots, highlighting the relationship between hypothyroidism-associated SNPs and ischemic stroke. Forest plots further facilitated the exploration of potential multiplicity and heterogeneity. While examining the symmetry in the funnel plots, an assessment of the stability of the results was attainable. In the final stage of the analysis, the results yielded from the IVW method were meticulously scrutinized through the employment of multinomial residuals and outliers, utilizing the MR-PRESSO (13) technique. This specialized model underwent careful examination and calibration, particularly with respect to horizontal multinomials and outliers, to ensure a robust and precise interpretation of underlying relationships.

### Meta-analysis of MR results from different databases

To determine the causative link between hypothyroidism and ischemic stroke, we sourced and assessed data from separate outcome databases. The datasets for ischemic stroke and its variants (no controls excluded), along with cerebrovascular disease sequelae and its variants (no controls excluded), were derived from the FinnGens database. In contrast, data specific to cerebral infarction were obtained from the UKB. MR analyses were independently conducted for each database. Subsequent to these individual MR analyses, the results of the IVW method were aggregated into a meta-analysis. Based on the detected heterogeneity (*I*^2^), models for analysis were chosen, and a fixed-effects model was adopted when heterogeneity was below 50%, whereas a random-effects model was utilized if this metric exceeded the given threshold. This methodological approach allowed us to generate a more comprehensive and nuanced overall estimate by incorporating the inherent variations inherent in each study.

### Sensitivity analysis

To evaluate the potential for horizontal multiplicity, we used the MR-Egger intercept term test, which has *p* > 0.05, indicating that horizontal multiplicity is missing (13). Through the application of the Egger-intercept method, as referenced in prior research (14), the analysis provided evidence for the absence of lateral pleiotropy, a phenomenon that could have potentially skewed the results. The statistical insignificance marked by a *p*-value greater than 0.05 further substantiates this finding, reinforcing the robustness of the result and aligning with the expectations of the study’s hypothesis. The MR-PRESSO test, utilized to identify horizontal pleiotropy anomalies, produced a *p*-value above 0.05, further affirming the non-existence of horizontal pleiotropy and the presence of outliers. The influence of each SNP on the outcomes was evaluated by sequentially omitting individual SNPs using the “leave-one-out” method (15). Cochran’s *Q* test was employed to evaluate heterogeneity within singular genetic variance estimations, with *p* > 0.05, denoting an absence of heterogeneity. All analytical procedures were diligently executed in the R software environment, specifically 4.1.3 version, esteemed for reliability in statistical analyses. For the MR investigation, we employed the “Two-Sample-MR” and “MR-PRESSO” packages, both of which are recognized for their specialized functionalities tailored for genetic causal inference tasks. Additionally, the “Meta” package was judiciously employed to conduct the meta-analysis, streamlining the integration and synthesis of results across different studies and thereby enhancing the rigor and comprehensiveness of our findings.

## Results

### IVs selection

This study used two-sample MR analysis to investigate the direct influence of hypothyroidism on susceptibility to ischemic stroke and its manifestations such as cerebral infarction and other cerebrovascular disease consequences. Ultimately, 64 SNPs were used as IVs for hypothyroidism and ischemic stroke, 64 SNPs were used as IVs for hypothyroidism and ischemic stroke (without exclusion of controls), 66 SNPs were used as IVs for hypothyroidism and cerebral infarction, 64 SNPs were used as IVs for hypothyroidism and sequelae of cerebrovascular disease, and 64 SNPs as IVs for hypothyroidism and sequelae of cerebrovascular disease (without excluding controls).

### Genetic association with ischemic stroke

**Figure 2:**
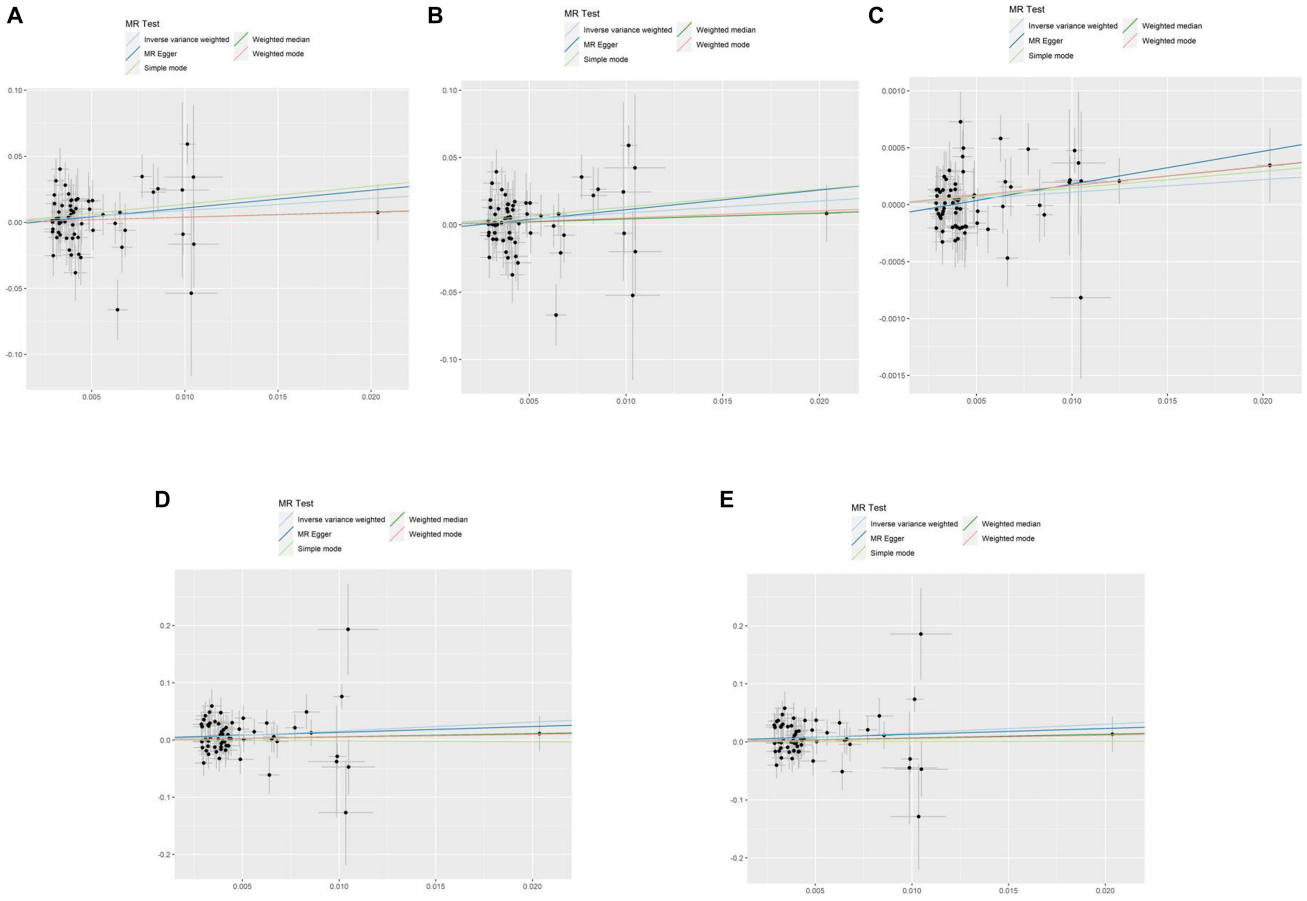
Scatterplots of the causal effect of hypothyroidism and ischemic stroke complications in primary MR. Analyses were conducted using IVW, MR-Egger regression, weighted median method, simple mode, weighted mode. The slope of each line corresponds to the estimated MR effect per method. IVW, inverse variance weighted; MR, Mendelian randomization; MR-Egger, MR-Egger regression. **(A)** Ischemic stroke; **(B)** ischemic stroke (no controls excluded); **(C)** cerebral infarction; **(D)** cerebrovascular disease sequelae; **(E)** cerebrovascular disease sequelae (without excluding controls).

**Figure 3:**
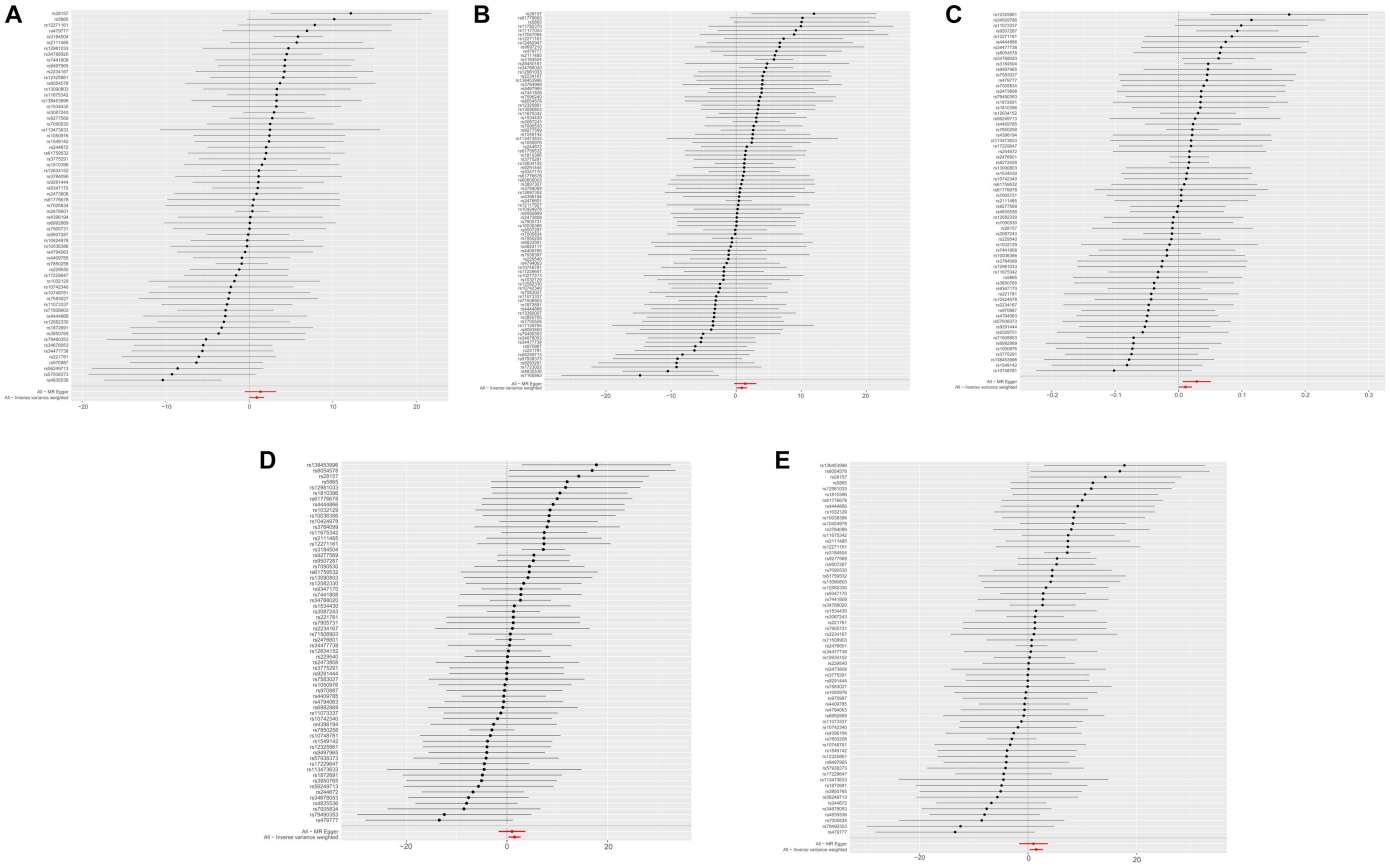
MR effect for hypothyroidism and ischemic stroke complications. MR, Mendelian randomization. **(A)** Ischemic stroke; **(B)** ischemic stroke (no controls excluded); **(C)** cerebral infarction; **(D)** cerebrovascular disease sequelae; **(E)** cerebrovascular disease sequelae (without excluding controls).

**Figure 4:**
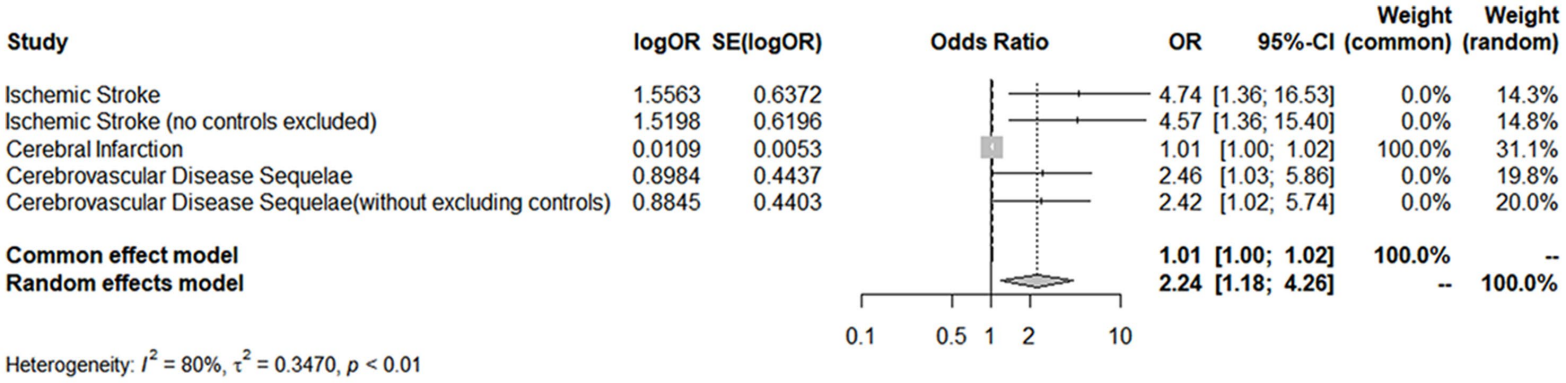
Meta-analysis of the MR results. MR, Mendelian randomization.

A comprehensive analysis was conducted using five different MR studies to examine the effects of hypothyroidism on five types of ischemic stroke. Using the scatter plots shown in Figure 2, we evaluated the direct causal relationship between hypothyroidism and various aspects of ischemic stroke (Table 2, Figure 3). The analysis illuminated a consistent and positive causal connection across all the investigations. Specifically, MR analysis exploring the relationship between hypothyroidism and ischemic stroke presented an OR of 4.7411 (95% CI:1.3598–16.5308, *p* = 0.0146) using the IVW method. The same relationship was further validated without excluding controls, resulting in an OR of 4.5713 (95% CI, 1.3570–15.3986; *p* = 0.0142). Additional MR analyses assessed correlations with specific manifestations, such as cerebral infarction (OR = 1.0110; 95% CI:1.0006–1.0215, *p* = 0.0373) and sequelae of cerebrovascular disease (OR = 2.4556; 95% CI:1.0291–5.8595, *p* = 0.0429). The latter was assessed in scenarios with and without controls, registering an OR of 2.4217 (95% CI,1.0217–5.7402, *p* = 0.0446). These findings further substantiate the underlying hypothesis, reinforcing the causal effect of hypothyroidism on the various dimensions of ischemic stroke. Intriguingly, with the exception of the MR outcome between hypothyroidism and cerebral infarction, the other four MR investigations indicated that the MR-Egger, WME, simple mode, and weighted mode analyses failed to reach statistical significance. The MR analysis results between hypothyroidism and cerebral infarction suggested that the MR-Egger and WME analysis results were in the same direction as the IVW analysis results, and none of the simple mode and weighted mode analysis results showed statistical significance. This discrepancy might stem from the inherent capabilities of the IVW methodology to handle variations in sample size, inherent statistical heterogeneity, or potential unidentified confounders. Meanwhile, a meta-analysis of the five MR study IVW outputs revealed pronounced heterogeneity (*I*^2^ = 80%, *p* = 0.001), prompting the utilization of the random-effects model. The statistical significance in the IVW results of the five MR analyses was confirmed by meta-analysis [2.24 (1.18–4.26), *p* = 0.025, Figure 4], following this adjustment. These findings substantiate the hypothesized existence of a causal effect, further supporting the conclusions drawn from the study. Despite the observed heterogeneity, potentially due to database variations, the overarching conclusions of the meta-analysis remained undeterred, thereby validating the conclusions of this study.

**Table 2:** Mendelian randomization (MR) analysis of hypothyroidism and ischemic stroke.

	MR methods	Hypothyroidism				
		NSNP	OR	95% LCI	95% UCI	*p*-value
Ischemic stroke	MR Egger	64	2.7782	0.1852	41.6648	0.4623
	WME	64	1.7491	0.2412	12.6844	0.5802
	IVW	64	4.7411	1.3598	16.5308	0.0146
	Simple mode	64	0.8646	0.0151	49.4311	0.9441
	Weighted mode	64	1.6305	0.1256	21.1708	0.7098
Ischemic stroke (no controls excluded)	MR Egger	64	2.7285	0.1961	37.9662	0.4578
	WME	64	1.9263	0.2427	15.2868	0.5350
	IVW	64	4.5713	1.3570	15.3986	0.0142
	Simple mode	64	1.0297	0.0211	50.2343	0.9883
	Weighted mode	64	1.7783	0.1275	24.8032	0.6700
Cerebral infarction	MR Egger	66	1.0293	1.0067	1.0523	0.0131
	WME	66	1.0169	1.0002	1.0339	0.0478
	IVW	66	1.0110	1.0006	1.0215	0.0373
	Simple mode	66	1.0147	0.9783	1.0525	0.4362
	Weighted mode	66	1.0171	0.9968	1.0377	0.1046
Cerebrovascular disease sequelae	MR Egger	64	3.8318	0.5808	25.2803	0.1678
	WME	64	1.4772	0.3706	5.8876	0.5803
	IVW	64	2.4556	1.0291	5.8595	0.0429
	Simple mode	64	3.9183	0.2345	65.4595	0.3454
	Weighted mode	64	1.4608	0.2222	9.6057	0.6946
Cerebrovascular disease sequelae (without excluding controls)	MR Egger	64	4.3035	0.6643	27.8790	0.1309
	WME	64	1.5474	0.3613	6.6273	0.5564
	IVW	64	2.4217	1.0217	5.7402	0.0446
	Simple mode	64	3.7511	0.2264	62.1629	0.3596
	Weighted mode	64	1.6789	0.3273	8.6116	0.5368

NSNP, the number of SNPs included in the MR analysis. MR, Mendelian randomization; SNPs, single nucleotide polymorphisms. OR, odds ratios (OR); 95% LCI, lower limit of 95% CI; 95% UCI, upper limit of 95% CI; IVW, inverse variance weighting; WME, weighted median method.

### Sensitivity analysis of MR

**Figure 5:**
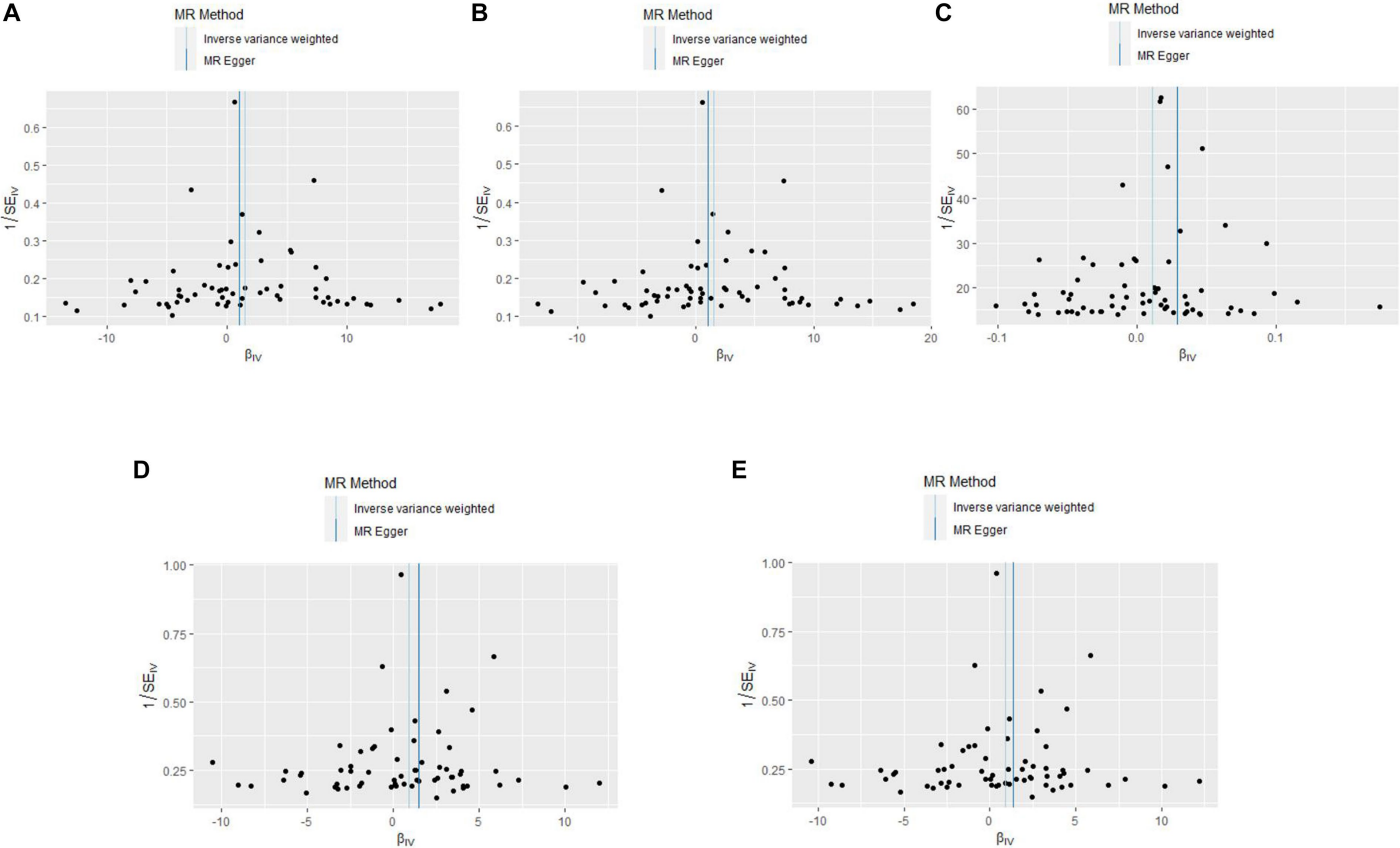
The funnel plots of MR Results between hypothyroidism and ischemic stroke. MR, Mendelian randomization. **(A)** Ischemic stroke; **(B)** ischemic stroke (no controls excluded); **(C)** cerebral infarction; **(D)** cerebrovascular disease sequelae; **(E)** cerebrovascular disease sequelae (without excluding controls).

**Figure 6:**
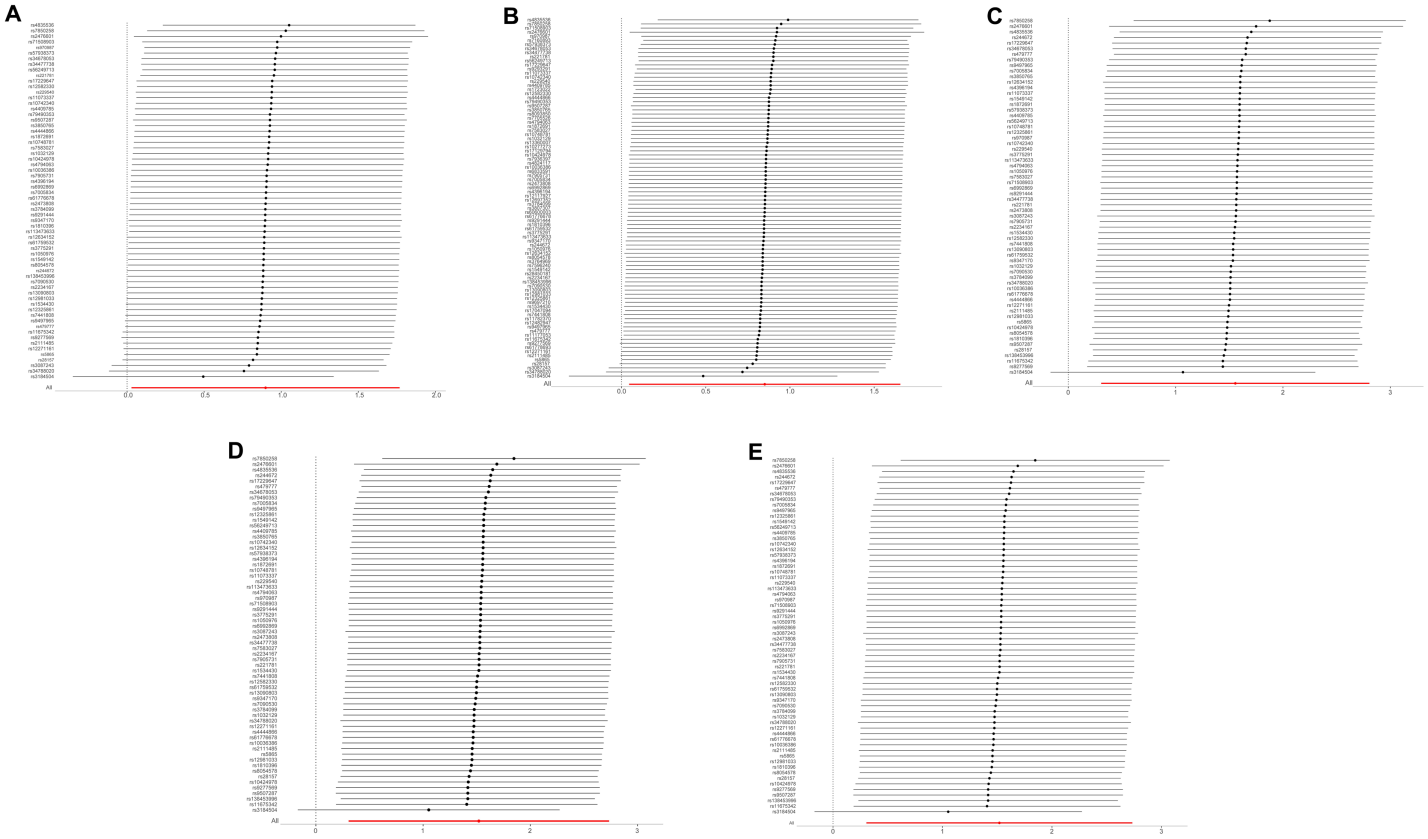
The “Leave-one-out” sensitivity analysis of MR Results between hypothyroidism and ischemic stroke. MR, Mendelian randomization. **(A)** Ischemic stroke; **(B)** ischemic stroke (no controls excluded); **(C)** cerebral infarction; **(D)** cerebrovascular disease sequelae; **(E)** cerebrovascular disease sequelae (without excluding controls).

The results of the five MR sensitivity analyses are shown in Table 3. The Cochran Q tests consistently yielded *p*-values greater than 0.05 for all the IVW outcomes, indicating an absence of heterogeneity in our MR investigations. The egger-intercept results, with *p* > 0.05, signify no evidence of horizontal pleiotropy in the study findings. Similarly, the MR-PRESSO results detailed in Table 4 display *p* > 0.05, indicating the absence of outliers and horizontal pleiotropy. A graphical representation in the form of a funnel plot, presented in Figure 5, shows a symmetrical distribution of causal effect estimates. This symmetry indicates an unbiased estimation, reaffirming the reliability of the SNPs used as IVs. Moreover, the resilience of our findings was verified through a “Leave-one-out” sensitivity analysis. During the “Leave-one-out” sensitivity analysis, it was discerned that the direct relationship estimations remained robust, unaffected by the inclusion or exclusion of any individual SNPs. This consistency was maintained even when each SNPs was sequentially omitted, as shown in Figure 6. All five results showed no heterogeneity or pleiotropy in the MR analysis between hypothyroidism and ischemic stroke.

**Table 3:** Sensitivity analyses for the causal results of the primary Mendelian randomization.

Outcome	MR methods	NSNP	Cochran *Q* statistic	*Q*_df	*Q*_*p*-value	MR-Egger intercept	SE	*p*-value
Ischemic stroke	MR Egger	64	70.2051	62	0.2219	0.0031	0.0072	0.6638
	IVW	64	70.4211	63	0.2433			
Ischemic stroke (no controls excluded)	MR Egger	64	67.2008	62	0.3036	0.003	0.007	0.666
	IVW	64	67.4047	63	0.329			
Cerebral infarction	MR Egger	66	60.6839	64	0.5945	−0.0001	0.0001	0.0784
	IVW	66	63.8829	65	0.5159			
Cerebrovascular disease sequelae	MR Egger	64	71.7387	62	0.1863	−0.0026	0.005	0.6037
	IVW	64	72.0537	63	0.2034			
Cerebrovascular disease sequelae (without excluding controls)	MR Egger	64	71.0925	62	0.2008	−0.0034	0.0049	0.4985
	IVW	64	71.624	63	0.2134			

NSNP, the number of SNPs included in the MR analysis. MR, Mendelian randomization; SNPs, single nucleotide polymorphisms; IVW, inverse variance weighting; MR-Egger, MR-Egger regression.

**Table 4:** MR-PRESSO for causal effect between hypothyroidism and ischemic stroke.

	MR methods	Hypothyroidism						
		NSNP	Beta	SE	OR	95% LCI	95% UCI	*p*-value
Ischemic stroke	MR-PRESSO	64	1.1875	0.5889	3.2787	1.0338	10.3985	0.315
Ischemic stroke (no controls excluded)	MR-PRESSO	64	1.1829	0.5733	3.2640	1.0611	10.0397	0.408
Cerebral infarction	MR-PRESSO	66	0.0116	0.0052	1.0116	1.0013	1.0220	0.256
Cerebrovascular disease sequelae	MR-PRESSO	64	0.6435	0.4250	1.9032	0.8274	4.3777	0.128
Cerebrovascular disease sequelae (without excluding controls)	MR-PRESSO	64	0.6052	0.4234	1.8315	0.7987	4.1999	0.125

NSNP, the number of SNPs included in the MR analysis. MR, Mendelian randomization; SNPs, single nucleotide polymorphisms; OR, odds ratios (OR); 95% LCI, lower limit of 95% CI; 95% UCI, upper limit of 95% CI; MR-PRESSO.

## Discussion

In the current study, the potential direct relationship between hypothyroidism and ischemic stroke was investigated using MR analysis. The MR analysis included five different outcome variables, and a comprehensive meta-analysis revealed a significant positive correlation between hypothyroidism and ischemic stroke.

Ischemic strokes is a notable factor that contributes to global morbidity and mortality. Ischemic stroke arises from the disruption of cerebral blood flow due to thrombosis or embolism, which leads to hypoxia and nutrient deprivation in the brain tissue, in turn causing severe neurological disability (16, 17). Its main etiological factors include endothelial dysfunction and atherosclerosis. Hypothyroidism is characterized by the thyroid gland’s inability to produce adequate thyroid hormone, essential for meeting the body’s metabolic needs. Hypothyroidism leads to a variety of clinical signs and symptoms, which are frequently nonspecific. Due to significant clinical variability, its diagnosis predominantly relies on biochemical markers. Overt hypothyroidism is characterized by elevated TSH and low free thyroid hormone levels, while mild or subclinical forms present with high TSH but normal free thyroid hormone levels (18). It has been suggested that DNA methylation of genes that regulate lipid concentrations *in vivo* may affect the evolution of ischemic stroke through fatty acid metabolic pathways and lipid modulation (19, 20). Furthermore, hypothyroidism and subclinical hypothyroidism may promote increases in low-density lipoprotein (LDL) (21) and total cholesterol (TC) levels. Such an elevation can exacerbate blood pressure (22), foster the development of insulin resistance, and ultimately induce obesity (22, 23). As a result, all of the above factors may increase the risk of atherosclerosis and impact stroke incidence. A meta-analysis demonstrated that for subjects with AIS, compared to those with normal levels of triiodothyronine (T3), patients with low T3 showed significant stroke severity in terms of NIHSS scores (MD = 3.18; 95% CI = 2.74–3.63; *I*^2^ = 61.9%) (24). In a substantial number of retrospective studies, the conspicuous impact of diminished serum T3 levels on the functional or recovery prospects of individuals suffering from AIS has been corroborated, with a particular emphasis on the elderly population (25, 26). A strong association was observed between reduced levels of free triiodothyronine (FT3) at the time of admission and unfavorable outcomes, as demonstrated by OR of 0.348 and a 95% CI of 0.18 to 0.72 (*p* = 0.007) (27). Furthermore, elevated TSH levels may be associated with higher mortality (28) and depression at the time of admission in individuals afflicted with stroke (29). In contrast, a meta-analysis revealed no substantial correlation between TSH concentration and ischemic heart disease or cerebrovascular events (30). These findings indicate a potential connection between untreated subclinical hypothyroidism and stroke events, although this association was not significant (29). These results contradict the findings of the previous studies. This discrepancy may be attributable to the impact of confounding variables and limited sample sizes in conventional observational investigations. However, the current MR study mitigates the influence of these confounding factors by utilizing large-scale GWAS data, thereby bolstering the confidence in the results.

The effects of hypothyroidism on ischemic stroke can be attributed to several potential mechanisms. First, the activation of NOD-like receptor thermal protein domain-associated protein 3 (NLRP3) inflammasomes may be intimately linked to the homeostatic imbalance of thyroid hormone (TH) (31, 32). Concurrently, studies have demonstrated that T3 treatment curbs hypoxia-induced DNA methylation and apoptosis, diminishes histone modifications, and provides protection to primary cortical neurons in hypoxic environments (33–35). In addition, T3 regulates various mechanisms of neuronal plasticity (36), stimulates astrocyte fatty acid oxidation, and augments neuroprotection and functional outcomes after stroke (37). Serum FT3 levels less than 4.38 pmol/L acted as a substantial prognostic indicator (sensitivity:78%; specificity:73%; AUC:0.77) (27). A comprehensive retrospective study illustrated that both hyperthyroidism and hypothyroidism might elevate the risk of stroke (38) and identified a unique inverted U-shaped correlation between high or low FT4 levels and overall cerebral perfusion (39) (nonlinear *p* = 0.002, correlation *p* = 0.024). This correlation suggests that hypothyroidism might amplify the risk of stroke via suboptimal cerebral circulation (39, 40). Based on MR analysis outcomes, hypothyroidism may potentially increase the risk of ischemic stroke. Therefore, vigilant monitoring of thyroid function may offer an effective preventive strategy against ischemic stroke.

This study provides genetic evidence to confirm that hypothyroidism increases the risk of ischemic stroke, as ascertained through two-sample MR analysis. This study has several strengths. First, it serves as a trailblazer in MR analysis. Extensive GWAS data were utilized in this innovative approach. This study aimed to examine the causal link between hypothyroidism and ischemic stroke. Second, MR analysis is effective in diminishing the impact of confounding factors by leveraging genetic variants that are constant and largely independent of environmental influences. Finally, MR analysis addresses the issue of reverse causation found in observational studies, effectively sidestepping the effects of reverse causation due to the unidirectional influence of genetic variants on phenotypes.

Nevertheless, our investigation possesses certain limitations. First, the results may not be generalizable to other populations as the study sample was exclusively drawn from Europe. Second, despite our best efforts to mitigate it, we cannot completely rule out the presence of pleiotropic factors such as horizontal pleiotropy, which could potentially bias the causal inference between hypothyroidism and ischemic stroke. More extensive research is necessary to confirm the validity of our findings.

## Conclusion

Our study identified a direct link between hypothyroidism and ischemic stroke, which suggests that hypothyroidism is a potential risk factor. Further comprehensive research is needed to elucidate the complex underlying biological mechanisms that connect these two conditions.

## Data availability statement

The original contributions presented in the study are included in the article further inquiries can be directed to the corresponding author.

## Author contributions

YT: Writing – original draft, Writing – review & editing. XS: Methodology, Writing – review & editing. JS: Data curation, Methodology, Writing – review & editing. XL: Methodology, Writing – review & editing. YB: Methodology, Writing – review & editing. LY: Methodology, Writing – review & editing. YL: Methodology, Writing – review & editing.

## Footnotes

^1^ https://www.ukbiobank.ac.uk/

^2^ https://r9.finngen.fi/
